# Spreading and Drying Dynamics of Water Drop on Hot Surface of Superwicking Ti-6Al-4V Alloy Material Fabricated by Femtosecond Laser

**DOI:** 10.3390/nano11040899

**Published:** 2021-04-01

**Authors:** Ranran Fang, Zekai Li, Xianhang Zhang, Xiaohui Zhu, Hanlin Zhang, Junchang Li, Zhonglin Pan, Zhiyu Huang, Chen Yang, Jiangen Zheng, Wensheng Yan, Yi Huang, Valeriy S. Maisotsenko, Anatoliy Y. Vorobyev

**Affiliations:** 1 School of Optoelectronic Engineering, Chongqing University of Posts and Telecommunications, 2 Chongwen Road, Nanan District, Chongqing 400065, China; fangrr@cqupt.edu.cn (R.F.); LZK_KAI@163.com (Z.L.); s190402010@stu.cqupt.edu.cn (X.Z.); zxh15111976635@163.com (X.Z.); zhl755345869@163.com (H.Z.); whaleair@163.com (J.L.); zhengjiangen@cqu.edu.cn (J.Z.); yws118@gmail.com (W.Y.); huangy@cqupt.edu.cn (Y.H.); 2 School of Science, Chongqing University of Posts and Telecommunications, 2 Chongwen Road, Nanan District, Chongqing 400065, China; pzl15185309575@163.com (Z.P.); huangzhiyu1026@hotmail.com (Z.H.); y2379202627@163.com (C.Y.); 3 M-Cycle Corporation, 1120 Delaware St. #110, Denver, CO 80204, USA; valeriymaisotsenko@gmail.com

**Keywords:** femtosecond laser processing, nanostructures, microstructures, laser-induced periodic surface structures (LIPSS), wicking materials, super-hydrophilic materials, surface capillarity, cooling of electronics, Maisotsenko cycle, global warming

## Abstract

A superwicking Ti-6Al-4V alloy material with a hierarchical capillary surface structure was fabricated using femtosecond laser. The basic capillary surface structure is an array of micropillars/microholes. For enhancing its capillary action, the surface of the micropillars/microholes is additionally structured by regular fine microgrooves using a technique of laser-induced periodic surface structures (LIPSS), providing an extremely strong capillary action in a temperature range between 23 °C and 80 °C. Due to strong capillary action, a water drop quickly spreads in the wicking surface structure and forms a thin film over a large surface area, resulting in fast evaporation. The maximum water flow velocity after the acceleration stage is found to be 225–250 mm/s. In contrast to other metallic materials with surface capillarity produced by laser processing, the wicking performance of which quickly degrades with time, the wicking functionality of the material created here is long-lasting. Strong and long-lasting wicking properties make the created material suitable for a large variety of practical applications based on liquid-vapor phase change. Potential significant energy savings in air-conditioning and cooling data centers due to application of the material created here can contribute to mitigation of global warming.

## 1. Introduction

Recent trends in cooling data centers [1,2], heat dissipation in high-heat flux electronics [3], cooling supercomputers [4], spacecraft thermal management [5,6,7], water desalination [8,9,10], waste heat recovery [11,12,13,14], cooling batteries [15], energy-harvesting [16,17,18,19], aircraft anti-icing [20], and Maisotsenko cycle (M-cycle) technologies [21,22,23,24,25] necessitate the creation of advanced capillary materials with efficient wicking/super-hydrophilic functionality at elevated temperatures. In particular, there is a fast-growing demand for these materials in cooling high-heat flux 5G electronics and in creating the next generation of M-cycle air-conditioning systems that will consume less electric power than the traditional compressor coolers by a factor of 5–8 [21,26,27,28,29]. In view of the fact that the air-conditioning of buildings and cooling data centers consume about 15% of global electricity generation, the energy efficiency in these sectors is an important factor for mitigation of global warming through reduction of air pollution associated with electricity generation.

A typical wicking medium currently used in heat pipes for cooling electronics is a porous material (mesh or sintered powder), performance of which degrades at high heat fluxes because of insufficient liquid transport and large thickness of the wicking medium (>0.5 mm) that limit the heat transfer [30,31,32]. Hierarchical wicking materials based on the surface capillary effect offer significantly enhanced cooling performance due to a high velocity of liquid transport, small thickness (<100 µm), and efficient evaporative functionality [33,34,35,36,37,38]. Prior studies also show that as compared with wire meshes, sintered powders, and microgroove structures, the micropillar arrays [39,40], especially hierarchical ones [41,42], provide superior heat transfer performance due to their good wicking, large surface area enhancement, and high evaporation rate owing to a large thin-film evaporation area [41,43,44]. Previously, surface nano/microstructuring of materials [34,45] using direct laser ablation [46,47,48,49,50] has been successfully applied to fabrication of superwicking silicon [36,51], metals [10,14,33,37,52], glasses [53,54], and polymers [38] by producing an array of parallel microgrooves on a surface of a material. It has been demonstrated that this open capillary surface structure provides a strong capillary force capable of unidirectional transporting of water for a long distance, even against the force of gravity. In contrast to previously studied microgroove structures produced by femtosecond laser processing, we investigate a hierarchical micropillar/microhole array structure. To enhance capillary action, the surface of micropillars/microholes is structured with regular fine microgrooves using a technique of laser-induced periodic surface structures [34,55,56]. In our work, the capillary structure is produced on a surface of a Ti-6Al-4V alloy sample. To characterize wicking functionality of the created material, we investigate water spreading distance *z* as a function of time *t* in a temperature range between 23 and 80 °C using high-speed video imaging. Our study shows that in the initial stage of the capillary flow, the water spreading distance achieves a very large value of about 9–11 mm at *t* = 100 ms and the maximum spreading velocity reaches about 225–250 mm/s in the studied temperature range, demonstrating excellent wicking performance at elevated temperatures.

Metals are preferable wicking materials for heat/mass exchangers due to their high inherent thermal conductance. However, previous studies have revealed that the creation of long-term stable metallic wicks encounters a problem of quick degradation of their capillary functionality caused by contamination via adsorption of hydrocarbons from the ambient air [57]. For example, femtosecond laser-treated metals are super-hydrophilic/superwicking immediately after laser processing, but they quickly become superhydrophobic after exposing to laboratory air from several days to several months [45,58]. Here, we find that in contrast to pure Ti [58] and other metals [45], the super-hydrophilic/wicking properties of titanium alloy (Ti-6Al-4V) degrade very slowly after femtosecond laser processing, making the created material suitable for practical applications. Due to the long-term stability and excellent wicking performance in a wide temperature range, the created material can significantly enhance the efficiency of commercially available M-cycle air-conditioners, where thick porous wicking cellulose pads are currently used. In particular, the application of the wicking material created here can provide significant energy savings in air-conditioning of buildings [59] and cooling data centers [60].

## 2. Experimental: Fabrication and Characterization

**Figure 1:**
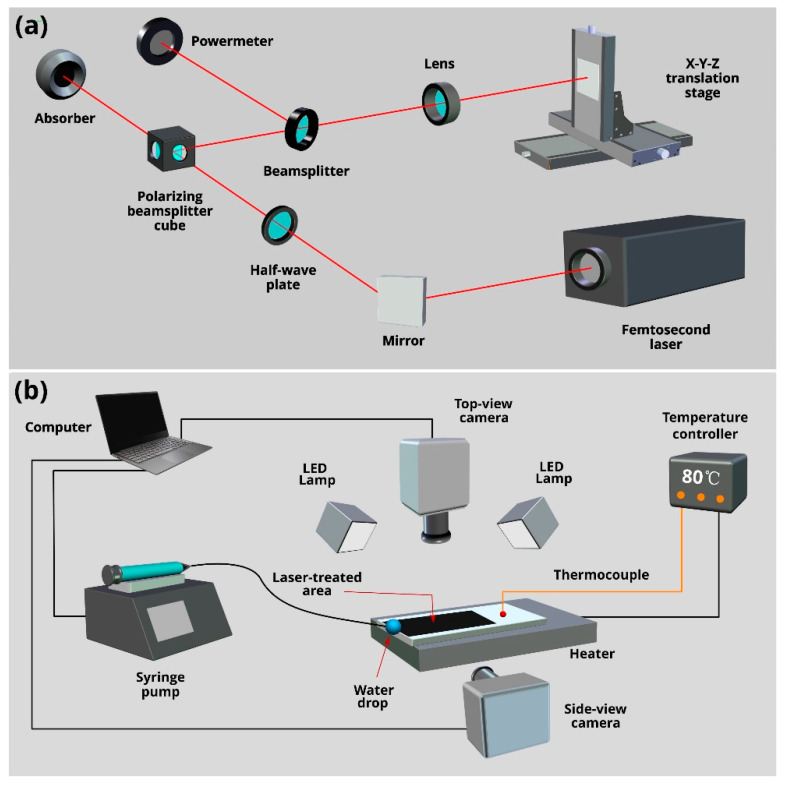
(**a**) Femtosecond laser setup for producing an array LIPSS-structured micropillars/microholes. (**b**) Experimental setup for high-speed video capturing of water spreading on the wicking surface at various temperatures.

In our study, we use Ti-6Al-4V alloy plates purchased from Goodfellow. Before laser processing, the samples were wiped with alcohol and then cleaned in an ultrasonic cleaner with distilled water at 40 °C to rinse away remaining contaminants. To fabricate an array of LIPSS-structured micropillars/microholes, we use a femtosecond laser processing setup, as shown Figure 1a. Our femtosecond laser system (Astrella, Coherent Inc., Santa Clara, CA, USA) generates 86 fs pulses with energy of 7.13 mJ/pulse at a maximum repetition rate of 1 kHz, with a central wavelength of 800 nm. A lens with a focal distance of 200 mm focuses the laser beam onto the Ti-6Al-4V sample mounted on a computer-controlled XYZ translation stage. To vary the laser power, a half-wave plate and polarizing beam-splitter cube are used. Measurements of the laser power are carried out using a non-polarizing beam-splitter and power meter. To produce the array of micropillars/microholes, we first fabricate an array of parallel microgrooves using a raster scanning of the sample across the laser beam. Then, we produce the second array of parallel microgrooves superimposed orthogonally onto the first one, resulting in an array of LIPSS-textured micropillars/microholes [56]. By varying laser fluence, step between scanning lines, scanning speed, and pulse repetition rate, we find laser processing parameters for producing a highly efficient wicking micropillar/microhole array structure. In this work, we fabricate a wicking array of LIPSS-structured micropillars/microholes at normal incidence of the laser beam using laser fluence of 5.1 J/cm^2^, laser spot diameter of 120 µm, step between scanning lines of 100 µm, pulse repetition rate of 1000 Hz, and scanning speed of 0.9 mm/s. Laser processing of Ti-6Al-4V alloy plates is carried out in air of atmospheric pressure at the temperature of 23 °C and relative humidity of 50%. The dimensions of the laser-textured surface area are 14 × 25 mm. The structural features of the produced wicking micropillar/microhole array are examined using a scanning electron microscope (SEM) MIRA 3 from Tescan (Brno, Czech Republic) and three-dimensional (3D) laser scanning microscope VK-X1100 from Keyence. The elemental composition of both treated and untreated sample surfaces is studied by energy dispersive X-ray spectroscopy (EDS) using a Brucker XFlash 6/30 detector (Karlsruhe, Germany). The wetting properties of the treated and untreated sample surfaces are characterized by measuring water contact angle, *θ*_CA_, using an OSA 200 system (Ningbo NB Scientific Instruments, Ningbo, China) equipped with an accessory for measuring *θ*_CA_ as a function of temperature, *T*.

The wicking properties of the produced structure are studied by video capturing the capillary flow dynamics of de-ionized water on a horizontally positioned sample using the experimental setup presented in Figure 1b. The studied sample is attached onto a heater. To regulate the sample surface temperature, we use a temperature controller (TCN4S from Autonics, Busan, South Korea) and thermocouple (5TC-TT-K-30-36 from Omega, Norwalk, CT, USA) mounted on the untreated sample surface. In our study, water is supplied to the capillary structure from a pendant 5 µL drop. To produce the pendant drop, we use a syringe pump Elite 11 from Harward Apparatus Inc. (Holliston, MA, USA). The water capillary flow dynamics are studied using a high-speed VEO 710L Phantom camera at a speed of 1000 frames per second (fps). The water flow behavior is investigated at sample surface temperature *T* = 23, 40, 60, and 80 °C, relative humidity of the ambient air of 50%, and ambient air temperature of 23 °C. Due to evaporation, the temperature of a pendant water droplet before its relocation onto the sample can be lower than the room temperature (23 °C). To measure the water droplet temperature, we use a miniature thermocouple placed inside the water droplet [61,62], and the water droplet temperature is measured to be 20.8 °C. From video recording, we find the dependence of water spreading distance, *z,* as a function of time, *t*. Using *z*(*t*) data, we also derive the water spreading velocity, *v,* as a numerical derivative ∆*z*/∆*t*, where ∆*z* is the difference of spreading distance between two consecutive video frames and ∆*t* = 10^−3^ s. In our study, we shot three videos for each studied temperature. After processing these videos, the most characteristic one was selected for presenting in this paper. In our study, we also investigated the water film thickness dynamics using a side-view camera (see Figure 1b). For side-view imaging, we used a high-speed V2012 Phantom camera.

## 3. Results and Discussion

**Figure 2:**
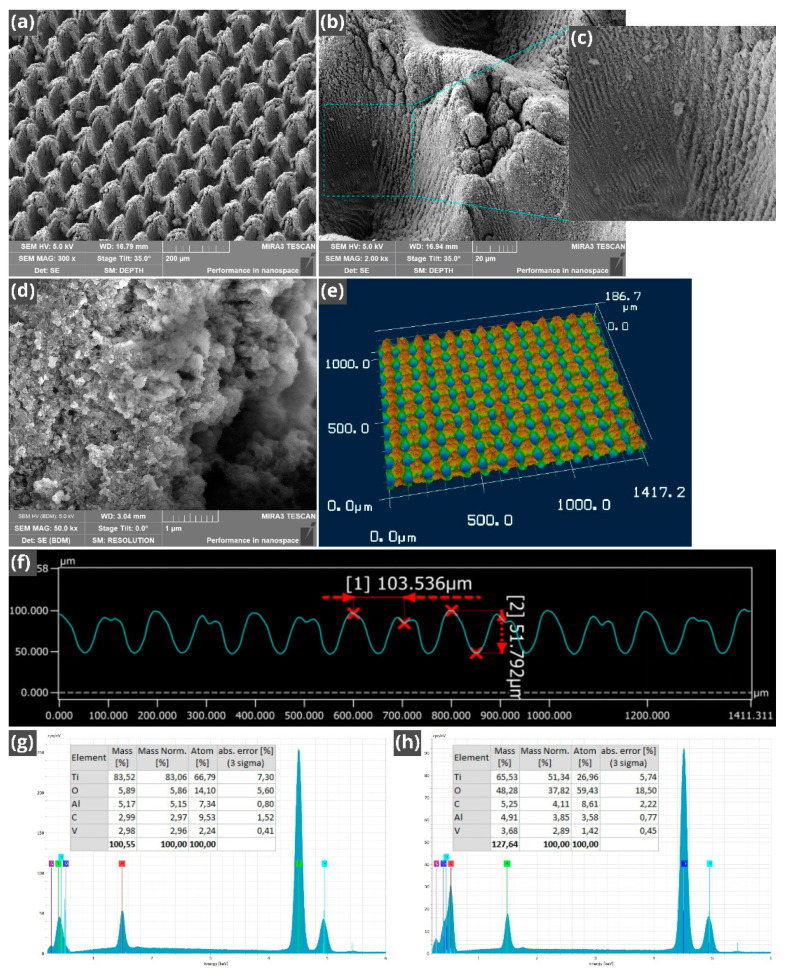
(**a**) Scanning electron microscope (SEM) image of the array of micropillars/microholes produced by femtosecond laser. (**b**) SEM image of a LIPSS-structured micropillar/microhole. (**c**) Magnified SEM image of LIPSS produced on micropillar/microhole walls. (**d**) SEM image of nanostructural features on the micropillar top. (**e**) Three-dimensional (3D) optical image of the array of micropillars/microholes. (**f**) Profile of the micropillars. (**g**) Elemental composition of the sample surface before laser processing. (**h**) Elemental composition of the sample surface after laser processing.

The commonly termed ‘laser technique for producing micropillars’ [56] produces both micropillars and microholes formed at intersections of the orthogonal scanning laser beams, thus giving rise to a hybrid structure of micropillars and microholes, where the micropillar bottom is taken as a base level. The structural features of the fabricated array of LIPSS-structured micropillars/microholes are shown in Figure 2a–f. The surface of micropillars and microholes is textured with both LIPSS and random fine nano/micro-structures of various shapes and dimensions (see Figure 2b–d). A 3D optical image of the array of micropillars/microholes is demonstrated in Figure 2e. The period and averaged height of the micropillars measured by 3D laser scanning microscope are 100 and 52 µm, respectively (see Figure 2f). The period and averaged depth of the microholes are 100 and 41 µm, respectively. The dimensions of the LIPSS and fine random nano/microstructures are in a range between about 50 nm and 10 µm (Figure 2b–d). The presence of LIPSS that partially covers the micropillar/microhole walls is an important structural feature. These periodic fine microgrooves enhance the overall capillary action of the fabricated wicking material through spreading water on the micropillar/microhole walls. As seen in Figure 2c, their period and width are about 1–3 and 0.5–1.5 µm, respectively. These periodic structures are a type of LIPSS produced by femtosecond laser pulses in multi-pulse ablation [34,55,63]. At a linearly polarized laser light, the LIPSS period, *d,* on a metal in an ambient dielectric medium reads [34]:

(1) d=λlas/(Re[η]∓sinθ) with g ‖ E

where *λ_las_* is the wavelength of incident laser light, *θ* is the angle of laser beam incidence, $\eta ={\left[\varepsilon_{d}\varepsilon_{metal}/(\varepsilon_{d}+\varepsilon_{metal})\right]}^{1/2}$ is the effective refractive index of the dielectric–metal interface for surface plasmons, *ε_d_* is the dielectric function of the ambient dielectric medium, *ε_metal_* is the dielectric function of the metal, Re[*η*] is the real part of *η*, **g** is the grating vector of LIPSS, and **E** is the tangential component of electrical field vector of the incident laser beam. Typically, *η ≈* 1 for air–metal interfaces when a metal surface is smooth [63,64,65]. Multi-pulse femtosecond ablation at low laser fluences gives rise to the formation of surface nanostructures that cause *η* to increase [63,64,65]. For example, *η ≈* 1.3 has been reported for LIPSS formation on Ti [64]. The increase in *η* results in LIPSS with a period smaller than the laser wavelength at *θ* ≈ 0. However, the increase in *θ* causes the LIPSS period to increase, giving rise to the LIPSS with *d* > *λ_las_*. The LIPSSs with the period larger than the laser wavelength have been previously studied in References [65,66]. It is known that LIPSS are produced by multi-pulse ablation of metals at laser fluence slightly above the damage threshold of a metal [34,55,63,65]. In the course of the micropillars/microholes fabrication, the laser-irradiated surface area significantly increases due to formation of surface structures, resulting in a reduction of laser fluence and creating conditions for LIPSS formation. Another factor that promotes LIPSS formation is a lower laser fluence on the laser beam periphery. Formation of the micropillars/microholes also causes the incidence angle to increase, resulting in increasing *d*. Here, we use these effects for producing LIPSS on the micropillar/microhole walls by a proper choice of laser processing parameters. An important feature of this approach is that this hierarchical capillary structure is produced by a one-step fabrication process. At the incidence angle of the laser beam between 30° and 80°, the LIPSS period for metals is typically in a range of 1–4 µm [65] that agrees with the range of *d* for the fine periodic microgrooves observed on the micropillar/microhole walls in our study. Previously, it has been shown that the surfaces of metals and Ti-6Al-4V alloy undergo a significant femtosecond laser-induced oxidation [67,68,69]. Therefore, after laser processing of the sample, we performed an EDS examination of the elemental composition of the laser-treated surface. This study shows a significant increase in the oxygen amount caused by femtosecond laser-induced oxidation, as seen in Figure 2g, h. It is known that TiO_2_ oxide has very good hydrophilic properties [57], therefore its formation caused by laser processing can be beneficial to super-hydrophilic/wicking properties of our sample.

The flow of a liquid in a capillary medium undergoes a sequence of regimes, including acceleration (*z* ∝ *t*^2^) [70,71,72], inertial (*z* ∝ *t*) [70,71,72,73,74,75], visco-inertial [36,76], classic Washburn’s regime (*z* ∝ *t*^1/2^) [77], and final stages [78]. In contrast to many previous works focused on the classic Washburn regime and other capillary flow stages occurring on the sub-second (0.1–1 s) and second (1–100 s) time scales [78,79,80,81,82,83,84,85,86,87,88,89,90,91,92,93,94,95], our study mainly focuses on the early capillary flow regimes on the millisecond time scale between 0 and 100 ms, understanding of which has recently become an important issue in fast remediation of dry-out spots in cooling high-heat flux electronics of 4G/5G telecom networks [96,97], Maisotsenko cycle (M-cycle) heat/mass exchangers [98,99,100,101], and miniaturization of microfluidic devices [102], where the classic Washburn regime and later capillary flow regimes do not occur because of the short length of capillary channels. The previous studies on the initial flow regimes mainly relate to the inertial regime in capillary tubes with a smooth surface, which are the simplest capillary systems [70,73,74,75]. Our capillary system has a high hierarchical structural complexity. Furthermore, due to water supply from a pendant drop, the forces driving the liquid are more complicated and include the capillary pressure due to surface structure, Laplace pressure from the curvature of the drop remaining between the sample edge and needle, Laplace pressure from the curvature of the drop located on the sample, and gravitational force of the drop on the sample surface [36,103,104]. Therefore, the inertial regime of water behavior in our capillary system may differ from that in the capillary tubes. As an example, the inertial regime is not observed in porous capillary media [74].

**Figure 3:**
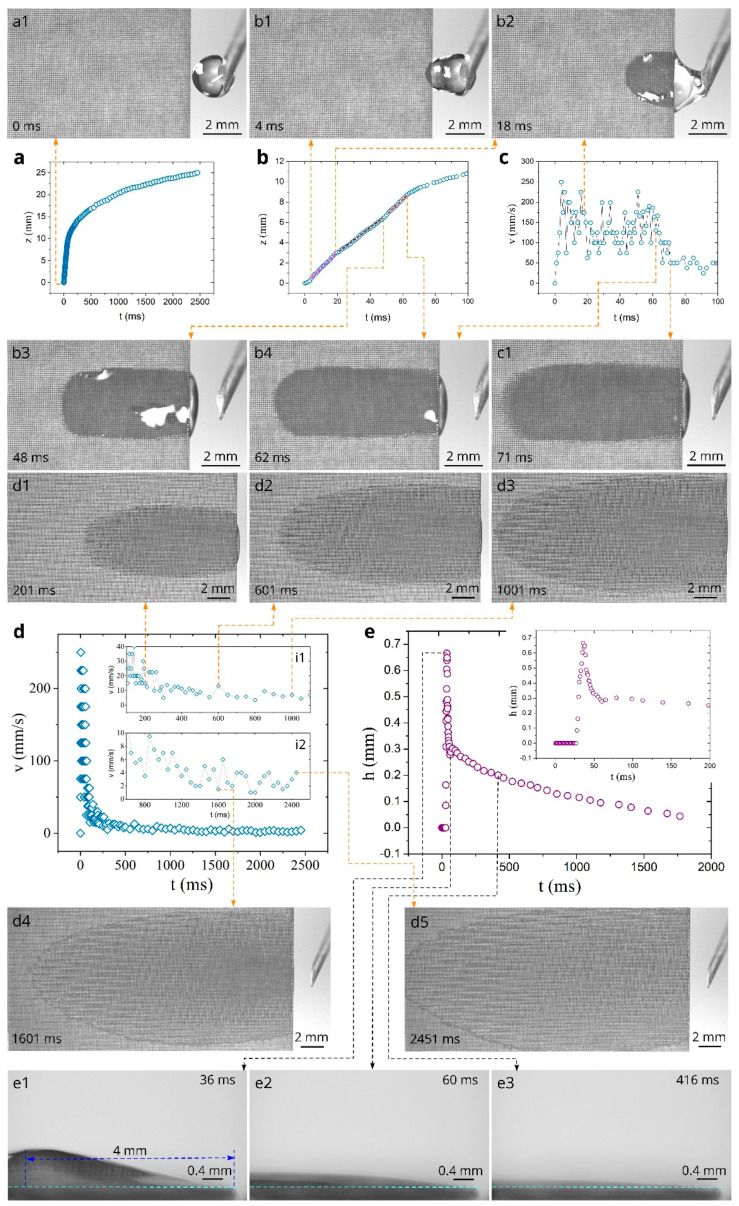
Dependences *z*(*t*), *v*(*t*), and *h*(*t*) at 23 °C and snapshots of water spreading (experiment was performed 72 days after laser processing of the sample). (**a**) The overall plot of the spreading distance as a function of time. (**b**) Detailed plot of spreading distance as a function of time between 0 and 100 ms. The dashed lines show three *z* ∝ *t* substages. (**c**) Plot of the velocity as a function of time between 0 and 100 ms. (**d**) The overall plot of the velocity as a function of time. (**e**) Plot of water film thickness as a function of time at *z* = 4 mm.

Figure 3a shows the overall plot of water spreading distance as a function of time at the sample temperature of 23 °C. It is seen that the capillary flow of water in the created material is very fast, and spreading distance reaches 25 mm in 2450 ms. Figure 3b presents the plot of spreading distance in the initial stages of water spreading in the time domain 0 < *t* < 100 ms, where one can see that the spreading distance achieves large values of about 6 and 11 mm at 45 and 100 ms respectively, demonstrating strong capillary action of the created material. The plot of capillary flow velocity as a function of time shown in Figure 3c reveals an acceleration stage between 0 and 4 ms, where the velocity increases from 0 to about 250 mm/s. Snapshot b1 in Figure 3b demonstrates water spreading in the end of the acceleration stage. As seen in Snapshot b2, about a half of the water drop is relocated from the needle to the sample at *t* = 18 ms. Snapshot b3 demonstrates that water relocation is almost completed at *t* = 48 ms. After the acceleration stage, in the time domain between 4 and 62 ms (see Figure 3b), the spreading distance exhibits quasilinear flow stage (*z* ∝ *t*) composed of three linear substages indicated by dashed lines. This water flow behavior can be explained by time-dependent contributions of the above-mentioned forces driving the liquid [36]. The time domains of the linear substages are 5–18, 20–48, and 51–62 ms. These time domains correlate with water drop spreading and its shape changes affecting the water driving forces, as seen in Snapshots b1–b4 and in Figure 3e, with its associated Snapshots e1–e3 that demonstrate dynamics of the water drop profile on the sample. The plot of the spreading velocity as a function of time presented in Figure 3c shows significant velocity fluctuations that are explained by an uncertainty in velocity derivation and pinning/depinning effects [36,38,105]. The average velocity in the quasilinear regime (4 < *t* < 62 ms) is found to be 142 mm/s. In the time domain 63 < *t* < 200 ms, the spreading velocity quickly decreases, reaching a value of about 20 mm/s at *t* = 200 ms. Snapshot c1 demonstrates water spreading in this time domain. At *t* > 200 ms, the velocity decreases slowly with smaller fluctuations (see Figure 3d and its insets i1, i2). It is seen from Snaphots d1–d5 that at *t* > 200 ms, the spreading water takes the shape of a thin film spread over a large surface area, indicating a potentially good evaporative functionality of the created material that is useful for applications based on liquid-vapor phase change. We also investigated the dynamics of water film profile using side-view video imaging of water spreading. We found that the water film thickness, *h,* achieves a maximum value at a distance of about 4 mm from the sample edge, and this position of maximum *h* almost does not change with time. The plot of the maximum *h* as a function of time at *z* = 4 mm is shown in Figure 3e. As seen in the inset of Figure 3e, the water spreading front reaches *z* = 4 mm at *t* = 28 ms, followed by a quick increase of the water film thickness that achieves a maximum value of 0.66 mm at *t* = 36 ms. Then, the water film thickness rapidly reduces to about 0.3 mm at *t* = 55 ms, indicating that the contributions to water flow of both Laplace pressure from the curvature of the drop located on the sample and gravitational force of this drop occur mainly in the time domain of the quasilinear inertial regime. At *t* > 55 ms, the water film thickness decreases slowly, and its value becomes 200, 100, and 50 µm at 420, 1100, and 1700 ms respectively, providing favorable conditions for efficient evaporation.

**Figure 4:**
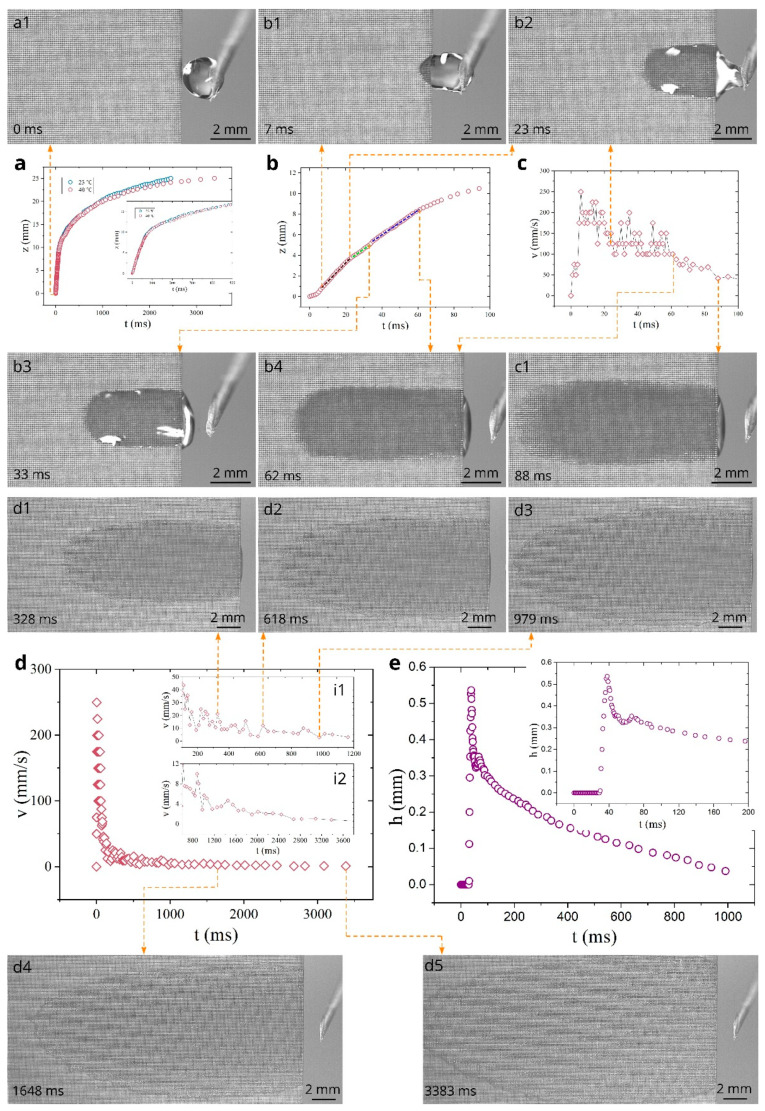
Dependences *z*(*t*), *v*(*t*), and *h*(*t*) at 40 °C and snapshots of water spreading (experiment was performed 74 days after laser processing of the sample). (**a**) The overall plot of the spreading distance as a function of time and its comparison with that at 23 °C. (**b**) Detailed *z*(*t*) plot between 0 and 100 ms. The dashed lines show three *z* ∝ *t* substages. (**c**) Plot of the velocity as a function of time between 0 and 100 ms. (**d**) The overall plot of the velocity as a function of time. (**e**) Plot of water film thickness as a function of time at *z* = 4 mm.

**Figure 5:**
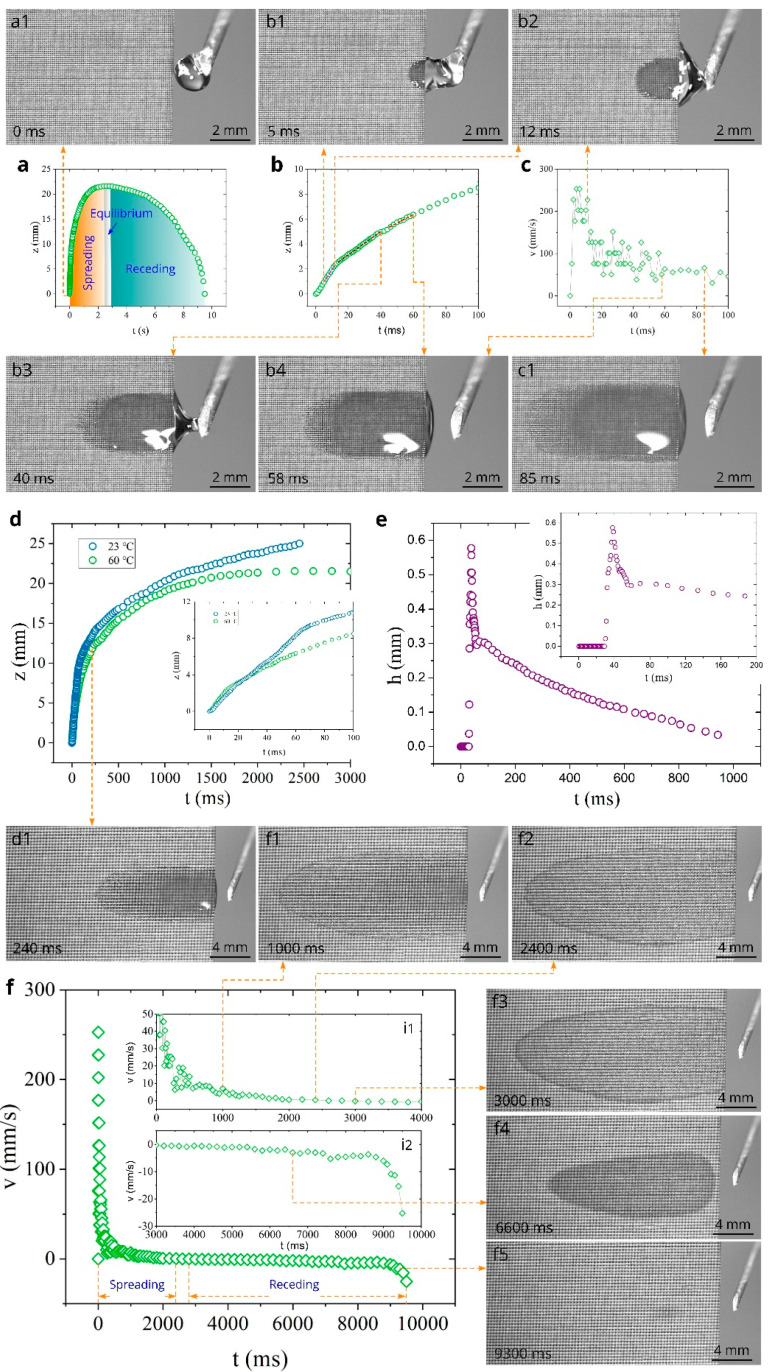
The *z*(*t*), *v*(*t*), and *h*(*t*) dependences and snapshots of water spreading and receding at 60 °C (experiment was performed 75 days after laser processing of the sample). (**a**) The overall plot of the spreading distance as a function of time. (**b**) Detailed *z*(*t*) plot between 0 and 100 ms. The dashed lines show three *z* ∝ *t* substages. (**c**) Detailed plot of the spreading velocity as a function of time between 0 and 100 ms. (**d**) The comparison of *z*(*t*) dependences at 23 and 60 °C. (**e**) Plot of water film thickness as a function of time at *z* = 4 mm. (**f**) The overall plot of spreading and receding velocities as a function of time.

The water spreading dynamics at 40 and 60 °C are presented in Figure 4 and Figure 5, respectively. Overall, the water behavior at 40 °C is similar to that at 23 °C, as seen from a comparison of Figure 4a with Figure 3a. In the initial stages, the *z*(*t*) plots at these temperatures are actually the same within the measurement uncertainty (see the inset in Figure 4a and compare Figure 4b with Figure 3b). Figure 4c shows that the acceleration stage takes place at 0 < *t* < 7 ms, resulting in a maximum velocity of about 250 mm/s. Similar to the water spreading at 23 °C, the spreading dynamics at 40 °C after the acceleration regime can also be characterized as a quasilinear regime composed of three linear substages, namely at 7 < *t* < 23 ms, 25 < *t* < 33 ms, and 35 < *t* < 62 ms, as shown by dotted lines in Figure 4b. Snapshots b1–b4 demonstrate the water spreading dynamics in this quasilinear regime. The *v*(*t*) dependence in the time domain 0 < *t* < 100 ms is presented in Figure 4c. This dependence is similar to that at 23 °C, as can be seen from the comparison of Figure 4c with Figure 3c. The average velocity in the quasilinear regime (7 < *t* < 62 ms) found from the data in Figure 4c is 144 mm/s, which is the same as at 23 °C within the experimental uncertainty. As seen in Figure 4a, the *z*(*t*) curves at 23 and 40 °C begin to divert at about 600 ms, revealing somewhat smaller spreading distance at 40 °C that can be explained by a higher evaporation rate with increasing temperature. Figure 4d and its insets i1 and i2 present both the overall *v*(*t*) dependence and detailed *v*(*t*) dependences at *t* > 100 ms, where it is seen that the velocity behavior is similar to that at 23 °C. Snapshots d1–d5 associated with the *v*(*t*) dependence in Figure 4d demonstrate water spreading dynamics at *t* > 328 ms. The plot of the water film thickness in Figure 4e also exhibits a similar behavior as at 23 °C. Thus, the presented experimental data show that the increase in temperature from 23 to 40 °C does not essentially affect the capillary water flow on the material created here.

Figure 5 shows both water spreading and receding (drying) dynamics at 60 °C. As seen in Figure 5a, the spreading distance achieves its maximum value of 21.5 mm at 2400 ms. This value is smaller than that at 23 °C (24.8 mm) and 40 °C (24.0 mm), indicating a noticeable effect of evaporation on the spreading distance. In the time domain 2400 < *t* < 2800 ms, the water-front actually does not move, revealing an equilibrium between the capillary and evaporation effects on water spreading. After the equilibrium stage, the water-front begins to recede with an increasing velocity, resulting in complete drying of the water film. The time domains of spreading, equilibrium, and receding stages of water behavior are indicated in Figure 5a. Figure 5b presents the *z*(*t*) dependence in the initial spreading stage between 0 and 100 ms, where three *z* ∝ *t* substages are marked with dashed lines. Snapshots b1–b4 demonstrate the water behavior in these substages. The velocity of capillary spreading in the initial time domain between 0 and 100 ms is shown in Figure 5c. It is seen that the water flow velocity remains to be high at 60 °C, achieving a maximum value of about 250 mm/s. The average velocity in the quasilinear regime (5 < *t* < 58 ms) is found to be 109 mm/s, which is smaller than that at 23 and 40 °C. Snapshots c1, d1, and f1 show the water film behavior after the quasilinear stage at 85, 240, and 1000 ms, respectively. Snapshot f2 shows the water film in the beginning of the equilibrium regime. The temperature effect on the water spreading behavior is demonstrated by a comparison of the spreading distances at 23 and 60 °C in Figure 5d, where the inset shows that the spreading distance at 60 °C becomes smaller already at about 30 ms. As seen in Figure 5e, the *h*(*t*) dependence does not undergo significant changes with increasing temperature to 60 °C. Figure 5f and its insets (i1 and i2) demonstrate the plots of both spreading and receding velocities in the entire lifetime of the water film on the sample surface. The receding regime begins at 2800 ms. Initially, the receding velocity increases slowly (see insets i1 and i2), achieving a value of 4.7 mm/s at 8800 ms. As seen in the inset i2, at *t* > 8800 ms, the receding velocity quickly increases and achieves a value of 25.3 mm/s at the end of the drying process. Snapshots f3, f4, and f5 show the water film drying dynamics. It is seen in Snapshot f5 that the evaporation of the water film comes to an end at *z* ≈ 4 mm, where the maximum water film thickness is observed.

**Figure 6:**
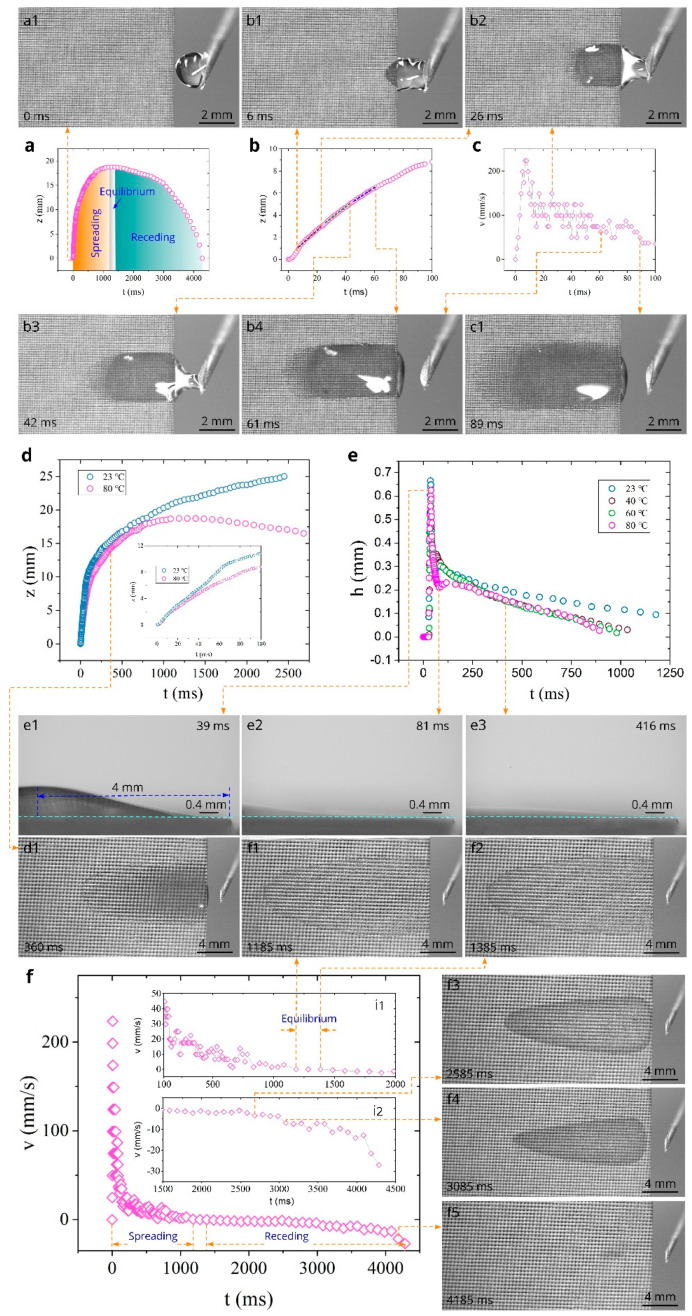
The *z*(*t*), *v*(*t*), and *h*(*t*) dependences at 80 °C and snapshots of water behaviors in spreading and receding regimes (experiment was performed 77 days after laser processing of the sample). (**a**) The overall plot of the spreading distance as a function of time. (**b**) Detailed *z*(*t*) plot in the time domain between 0 and 100 ms. The dashed lines show three *z* ∝ *t* substages. (**c**) Detailed plot of the spreading velocity as a function of time between 0 and 100 ms. (**d**) The comparison of *z*(*t*) dependences at 23 and 80 °C. (**e**) The comparison of *h*(*t*) dependences at the studied temperatures. (**f**) The overall plot of spreading and receding velocities as a function of time.

The data on water spreading and receding (drying) behaviors at 80 °C are presented in Figure 6. The overall *z*(*t*) dependence is shown in Figure 6a, where the time domains of spreading, equilibrium, and receding regimes are indicated. It is seen that the maximum spreading distance is observed to be 18.7 mm in the equilibrium stage between 1185 and 1385 ms, indicating a significant effect of evaporation on the maximum spreading distance. The detailed *z*(*t*) dependence in the time domain between 0 and 100 ms is demonstrated in Figure 6b, where three *z* ∝ *t* substages are marked with dashed lines. Snapshots b1–b4 associated with these substages show the water behavior in these linear substages. The *v*(*t*) plot in the time domain between 0 and 100 ms (Figure 6c) shows that the maximum capillary flow velocity remains high (about 225 mm/s). The average velocity in the quasilinear regime (6 < *t* < 61 ms) also remains high (108 mm/s), demonstrating excellent wicking properties at high temperatures. Snapshots c1 and d1 show the water film behavior in the spreading regime after the quasilinear stage. The temperature effect on the water spreading is clearly seen in Figure 6d, where a comparison of the *z*(*t*) plots at 23 and 80 °C is presented. The inset in Figure 6d shows that these plots begin to differ at about 20 ms. In the time domain *t* > 750 ms, the evaporation effect on the water behavior becomes very significant, resulting in the equilibrium and then in receding stages of water dynamics indicated in Figure 6a. Snapshots f1 and f2 show the water film in the beginning (*t* = 1185 ms) and end (*t* = 1385 ms) of the equilibrium regime. Figure 6e and Snapshots e1–e3 obtained by the side-view camera show the water film profile dynamics. The temperature effect on the *h*(*t*) dependence at *z* = 4 mm (location of the maximum water film thickness) is demonstrated in Figure 6e, where we also included the *h*(*t*) dependences at other studied temperatures. These data show that at *t* = 780 ms, the water film thickness decreases from 142 to 68 µm as the temperature rises from 23 to 80 °C. The overall plot of spreading and receding velocities as functions of time is presented in Figure 6f, where the time domains of the spreading, equilibrium (see the inset i1), and receding regimes are also shown. At *t* > 1385 ms (see the inset i1), the water film front begins to recede with an increasing velocity, achieving an extremely high value of 27 mm/s at 4185–4285 ms (see the inset i2). The sample surface becomes completely dry at 4285 ms. Snapshots f3–f5 demonstrate the evaporating water film behavior in the receding regime. Snapshot f5 shows that the water film evaporation terminates at *z* ≈ 4 mm, i.e., at the same location as at 60 °C (see snapshot f5 in Figure 5f).

**Figure 7:**
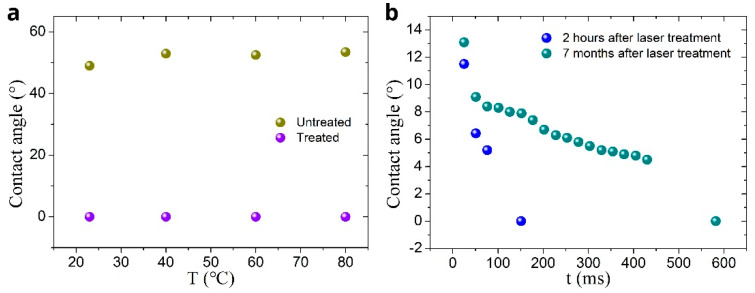
(**a**) Contact angle of a 2 µL water drop on untreated and treated sample surfaces as a function of temperature. (**b**) Contact angle dynamics of a 2 µL water drop on the treated sample measured immediately after laser processing and 7 months later.

The contact angle, *θ*_CA_, is an important parameter in the capillary flow [106]. Therefore, we measured *θ*_CA_ as a function of temperature for both untreated and treated surfaces. The obtained results are presented in Figure 7a. It is seen that in a temperature range between 23 and 80°, the contact angle of the untreated surface increases from 49 to 53.5°. The treated surface exhibits *θ*_CA_ ≈ 0° in the entire studied temperature range, demonstrating stable super-hydrophilic properties with the variation in temperature. As compared with other reported micropillars’ structures [107,108,109], our structure provides a better capillary performance. For example, a titanium micropillar array reported in Reference [107] demonstrates water spreading distance of about 5 mm within 100 ms for a water drop supply at the sample edge, whereas our structure transports water for the distance of about 11 mm (see Figure 3b).

A critical problem in creating capillary metallic surfaces is a quick degradation of their capillary performance caused by adsorption of hydrophobic hydrocarbons from the ambient air [45,57,58]. In our work, the superwicking functionality degradation over time was assessed by measuring the contact angle, *θ*_CA_, of a deposited water drop using the OSA 200 system in video recording mode at a speed of 40 fps, allowing to measure the contact angle as a function of time after the drop deposition on the laser-structured surface. This test over a time period of about 7 months shows that the water static contact angle remains to be close to zero. During this period, the sample used in our experiments was subjected to multiple heating/cooling cycles, multiple wetting/drying cycles, and long exposure to Lab air containing hydrocarbons due to the presence of a large number plastic items in the Lab. The results of the test on contact angle dynamics performed immediately after laser processing and 7 months later are shown Figure 7b. It is seen that immediately after laser processing, the contact angle of a water drop deposited on the sample becomes close to zero within about 200 ms, while 7 months later, this time is about 600 ms, indicating some degradation of the wicking action. Despite this degradation, our sample retains its super-hydrophilic/wicking properties (*θ*_CA_ ≈ 0°), in contrast to other laser-treated metals, which become superhydrophobic (*θ*_CA_ > 150°) in 2–3 months [45,56,58]. Taking into account that the number of wicking metallic materials with surface capillarity is currently very limited, the creation of both long-lasting and efficient wicking surface in our work is a significant result for further advancing important technological areas mentioned in the Introduction Section. An important feature of laser processing technologies is that modern industrial laser systems with a high pulse repetition rate (up to MHz-range) provide a laser nano/microstructuring speed up to about 1 m^2^ s^−1^, allowing mass production [55].

## 4. Conclusions

In this work, we fabricated an array of periodic micropillars/microholes on a surface of a Ti-6Al-4V alloy plate, using a femtosecond laser nano/microstructuring technique. For enhancing capillary action, the surface of micropillars/microholes was additionally structured with both laser-induced periodic surface structures and fine random nano/microstructures. The created hierarchical capillary surface structure exhibited an extremely good capillary functionality in the temperature range between 23 and 80 °C. After the acceleration stage, the maximum water flow velocity in the inertial regime was found to be very high, achieving about 250 mm/s. The strong capillary action provides fast water spreading for a long distance (up to 5.6–7.2 mm) even in the inertial flow regime (0 < *t* < 50 ms) at all studied temperatures. In a later stage of capillary flow, water quickly spread and formed a thin film over a large surface area, providing conditions for fast evaporation needed in applications based on liquid-vapor phase change. An important feature of the created material is a very slow degradation of its wicking functionality with time. The long-lasting wicking functionality along with a wide operational temperature range make the created texture suitable for a variety of practical applications, where the choice of materials is currently very limited, such as cooling high-heat flux electronics, thermal and fluid management in aerospace systems [5,6,7], cooling data centers [1,2], heat dissipation in power batteries and electronics of hybrid vehicles [15,110,111], and M-cycle applications, including air-conditioning [21,22,23,24,25], waste heat recovery [10,11,12], water desalination/purification [8,9,112,113,114], and cooling towers [115]. Potential significant energy savings in air-conditioning of buildings [59] and cooling data centers [60] due to application of the material created here can contribute to mitigation of global warming.

## Data Availability

Data are contained within the article.
